# Neutrophils: the forgotten cell in JIA disease pathogenesis

**DOI:** 10.1186/1546-0096-5-13

**Published:** 2007-06-13

**Authors:** James N Jarvis, Kaiyu Jiang, Howard R Petty, Michael Centola

**Affiliations:** 1 Department of Pediatrics, University of Oklahoma College of Medicine, Oklahoma City, OK 73014, USA; 2 Deparment of Ophthamology, University of Michigan School of Medicine, Kellogg Eye Institute, Ann Arbor, MI 48105, USA; 3 Arthritis & Immunology Program, Oklahoma Medical research Foundation, Oklahoma City, OK 73104, USA

## Abstract

Juvenile idiopathic arthritis (JIA) has long been assumed to be an autoimmune disease, triggered by aberrant recognition of "self" antigens by T-cells. However, systems biology approaches to this family of diseases have suggested complex interactions between innate and adaptive immunity that underlie JIA. In particular, new data suggest an important role for neutrophils in JIA pathogenesis. In this short review, we will discuss the new data that support a role for neutrophils in JIA, discuss regulatory functions that link neutrophils to adaptive immune responses, and discuss future areas of investigation. Above all else, we invite the reader to re-consider the use of the term "autoimmunity" as applied to the family of illnesses we collectively call JIA.

## Note on nomenclature

The nomenclature used to categorize children with chronic arthritis has undergone considerable change over the years covered by this review. The most widely accepted classification scheme replaces the terms juvenile *rheumatoid *arthritis and juvenile *chronic *arthritis with the term juvenile *idiopathic *arthritis. However, it is important for the reader to note that the terms are not synonymous and classification schemes do not completely overlap. This leaves the reviewer with the vexing task of how to refer to earlier studies that used different classification schemes. For the sake of simplicity, clarity, and accuracy, we have elected to use whatever terms were used by the authors of the relevant studies in describing those studies, while adhering to the internationally-accepted classification scheme when speaking generically of chronic arthritis in children.

## Background

With the possible exception of the systemic-onset form [1], juvenile idiopathic arthritis (JIA) has long been assumed to be an "autoimmune" disease. The autoimmune theory of pathogenesis has tenaciously held its position of unchallenged dogma in pediatric rheumatology, despite serious limits in its ability to explain all the known immunopathological phenomena and the paucity of evidence for either a T- or B-cell driven response (see below). At the same time, no competing theories for pathogenesis have emerged, so the autoimmune theory holds its ground by default. In this review, we will discuss emerging evidence that demonstrates the importance of neutrophils in regulating and informing the adaptive immune response, suggesting, therefore, that innate immunity may play a larger role in JIA pathogenesis than has been previously thought. We will then summarize new data from our laboratories that supports the hypothesis that neutrophil activation in JIA is a *primary *pathogenic event, and that involvement of the adaptive immune system is downstream of that primary event.

This paper is not intended as a comprehensive review of specific neutrophil function. Other authors have already taken on that daunting task [e.g.,[2-5]]. We will summarize relevant neutrophil biology in context, however, in appropriate places in this text.

## Limits of the T cell centered hypothesis

The presence of T cells within the inflamed synovium, the frequency of antinuclear antibodies, and the association of specific subtypes with specific HLA alleles [6] have all been powerful arguments that the illnesses collectively referred to as JIA represent an "autoimmune" disease whose pathogenesis is rooted in the dysregulation of mechanisms through which T cells distinguish "self" from "non-self" [7]. However, over the past 20 years, little additional evidence has accumulated to support T-cell centered theories of pathogenesis. Furthermore, our expanding knowledge has demonstrated equally plausible explanations for some of the phenomena on which the autoimmune hypothesis of JIA pathogenesis was built. For example, T cells can be found in joints of adults with gout [8,9] clearly demonstrating that the presence of T cells within a joint does not require autoimmune activation. Moreover, an increasing body of evidence demonstrates how T cells can be recruited to and activated in the synovial space [10-12] without engagement of the T cell-receptor-HLA complex, a process believed to be crucial in JIA pathogenesis. The pathological meaning of autoantibodies, and ANA in particular, in childhood arthritis becomes less significant as we have established that the presence of such autoantibodies is a common feature in childhood [13,14] and not necessarily a sign of dysregulated immunity. An additional Achilles heel of the autoimmune theory of JIA pathogenesis is the fact that HLA associations seen in one ethnic group are not found in others, suggesting that HLA is an associative, not mechanistic, linkage to JIA[15]. Finally, and perhaps most importantly, T cell-centered theories of JIA pathogenesis have not provided the foundation for a single new treatment for this disease. Current successful therapies are either non-specific (e.g., methotrexate) or target proteins that are critical elements of the innate immune response (e.g., TNFα). Thus, there is an urgent need to rethink the old paradigm and search more imaginatively for future targets of therapy.

### Neutrophils: seneschals of the adaptive immune response

Immunologists have long overlooked the neutrophil. Lacking the elegant specificity of T and B cells and their intricate "dance" with antigen processing cells and multiple co-receptors, the neutrophil, with its, prepackaged arsenal of inflammatory ordnance, short life span, and all-too-well-recognized capacity to injure host tissue [16], appeared to be the "loose cannon" of the immune system. A more sophisticated model of neutrophil action has evolved as we have come to understand better the complex interplay between the innate and adaptive immune systems.

Until recently, it was common to think separately about innate and adaptive immunity. We typically think of innate immunity as occurring early in an immune response, being non-specific in nature, and completed at the time specific, adaptive immune mechanisms come into play. Although this view has the virtues of simplicity and clarity (and thus provides a useful heuristic framework), the truth is that innate and adaptive immunity display numerous points of intersection and co-regulation [17,18].

Neutrophils provide one of the critical points of intersection between innate and adaptive immunity. Indeed, we are now learning that neutrophils, like complement, play a critical role in instructing and shaping almost every aspect of the adaptive immune response.

**Figure 1:**
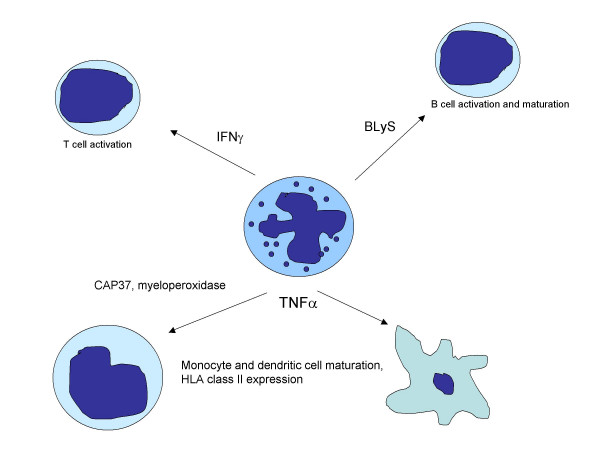
Interplay of innate and adaptive immunity showing multiple actions of neutrophils on the adaptive immune response. This figure is not intended to be comprehensive. Rather it shows in a highly-simplified fashion the role of neutrophils in informing the adaptive immune response and some of the mediators through which those effects are regulated.

Current states of knowledge suggest that an inflammatory response must begin with the immune system's sensing two classes of signals: tissue injury and the presence of a foreign genome [19]. The presence of only one is inadequate to begin the process. Surgery, for example, injures tissues, but excites a relatively modest inflammatory response because of its being performed sterilely; commensurate bacterial flora expose us daily to foreign genetic material, but elicit little inflammatory response in the absence of tissue injury [20]. Neutrophils play a critical role in sensing these binary signals, and, of most importance to our understanding of JIA pathogenesis, can serve as a *central *point of orchestration of subsequent *adaptive *immune responses. Upon sensing the binary signals that trigger the inflammatory process, neutrophils begin a program that determines whether there will be a brisk, modest, or blunted immunologic response (see Figure 1). Release of neutrophil myeloperoxidase [21] and anti-bacterial cationic proteins [22] up-regulates HLA class II molecules and induces chemotaxis in monocytes. Similar effects are induced on dendritic cells, mediated, in part, by neutrophil release of TNFα[23,24]. However, neutrophils' influence on adaptive immunity extends beyond the early phases of antigen-presenting cell activation and immune response induction. Neutrophils also release the TNF-related ligand, B lymphocyte stimulator (BLyS), thus regulating the expansion and maturation of B cells [25]. In a similar vein, neutrophil-derived IFNγ regulates the activation and expansion of T cells [26].

Thus, rather than being "downstream" of the putative pathological events in JIA, neutrophils are arguably at the very center. Furthermore, data from patients with known *autoinflammatory *syndromes clearly demonstrate that disrupted innate immunity can lead to clearly defined aberrations in adaptive immunity [27-29]. How, then, can the field of pediatric rheumatology avoid a detailed study of the neutrophil as we confront the limitations of T cell-centered theories of JIA pathogenesis? Isn't it time to look upstream? These are rhetorical questions; it should be obvious from the data that has emerged in the past 7 years that we no longer can avoid a systematic examination of neutrophil function in JIA, especially now that we have improved tools and methodologies for doing so. In all fairness, given the paucity of information that emerged from early studies of neutrophils in JIA, it is easy to see why this area was relatively neglected for so long.

### Neutrophils in JRA/JIA: early studies

Early interest in neutrophils derived from the fact that they are the most abundant cell within JRA synovial fluid [30]. Several early studies examined neutrophil chemotaxis, with some showing no differences when JRA neutrophils were compared with controls [31] and others showing increased motility of JRA neutrophils [32] although the increases were observed only during periods of active disease. There is a more extensive literature examining neutrophil function in adult rheumatoid disease, although they, too, provide conflicting data. Studies of circulating neutrophils from adults with rheumatoid arthritis have generally reported absence of markers associated with the activated state [33,34] (although there are conflicting data that suggest otherwise [35]) and normal chemotaxis [36] (although, again, data from different studies appear to conflict [37]). The applicability of these findings to childhood-onset disease is uncertain when thinking in traditional terms of neutrophil function. More recent studies suggest that these studies were inconclusive because neutrophils' *regulatory *potential may be more relevant than their *effector *functions in JIA pathology.

With so much conflicting data, it is easy to see why pediatric rheumatologists quickly dropped the neutrophil to explore other potentially more productive areas of immunology. By the 1990s, the neutrophil had all but disappeared from the pediatric rheumatology literature, with the exception of a single study from Poland [38], a study whose significance was unappreciated at the time. All of this changed seven years ago, as an understanding of neutrophils regulatory potential began to unfold.

### Evidence for chronic neutrophil activation in JIA/JRA

In 2000, Frosch and colleagues from the University of Munster in Germany reported their investigations with myeloid-related proteins (MRP-8 and -14) in juvenile rheumatoid arthritis [39]. These proteins are made by both monocytes and neutrophils and are released during leukocyte interactions with endothelium [40]. These authors reported a striking correlation between MRP-8 and -14 levels and disease activity in a cohort of children with pauciarticular JRA. While these authors assumed that monocytes were the cell of origin for the proteins, subsequent work from this same group (examining the related protein, S100 A12) attributed the abundance of S100 proteins in JRA sera to neutrophil activation [41]. Indeed, neutrophil (or monocyte)-derived S100 proteins are beginning to emerge as a potentially useful clinical biomarker to monitor response to therapy in children with JIA. At the risk of generating some healthy controversy, it is worth pointing out that no reliable T- or B-cell based biomarker for response to therapy in JIA has emerged in the past 20 years.

Before the publication of the Frosch and Foell papers, we had posited that JIA immune pathogenesis very likely involves complex interactions between innate and adaptive immunity [42] and ongoing work from our group has aimed at elucidating those interactions [43]. We have therefore undertaken a "systems biology" [44,45] approach to polyarticular JIA in an effort to obtain the "global view" of these interactions [46]. In a preliminary study, designed largely to develop methodologies [43], we examined gene expression profiles of whole blood buffy coats in a small group (n = 9) of children with active JRA. In that study, we were quite surprised to see over-expression of genes either expressed by neutrophils (e.g., ferritin heavy and light chains, N-formyl peptide receptor) or regulating neutrophil function (e.g., IL-8) in children with JRA. These data were consistent with the data emerging from the Frosch and Foell studies; neutrophils likely play an important role in JRA/JIA pathogenesis.

**Figure 2:**
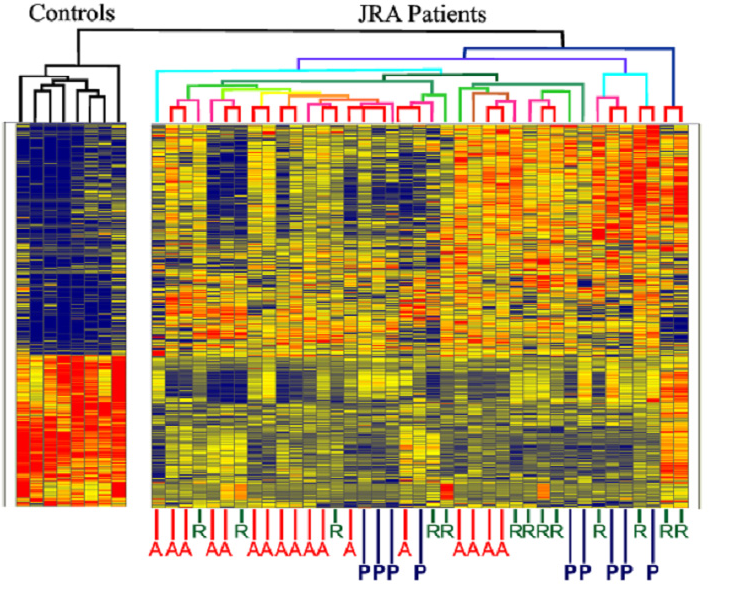
Hierarchical cluster analysis of gene expression profiles in children with polyarticular, RF-negative JRA. Gene expression profiles are compared with those of healthy controls, shown on the left of the grid. Children with active disease ("A") scatter through the heat map with children with "responsive" ("R" = "inactive") disease and children who have shown a "partial" response to therapy ("P" = have met ACR30 criteria). From Jarvis et al, *Arthritis Res Therapy *2006. The authors retain the copyright for this material.

**Figure 3:**
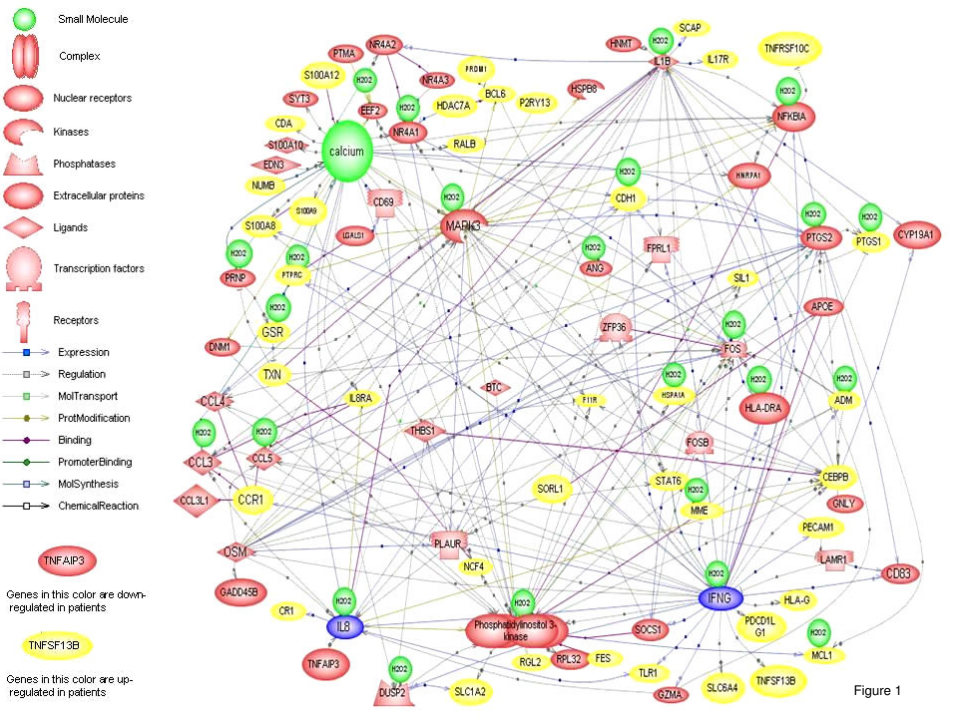
*In silico *modeling derived from Pathway Assist software analysis of differential gene expression in JRA neutrophils compared with healthy control subjects. Note clusters of IFNγ-(lower right) and IL-8- (lower left) regulated genes. From Jarvis et al, *Arthritis Res Therapy *2006. The authors retain the copyright for this material.

We next performed gene expression profiling on neutrophils of 25 children with JRA, 15 of whom were studied during periods of active and inactive disease as defined by Wallace and colleagues [47]. In that study, we demonstrated aberrant patterns of gene expression in neutrophils of children with polyarticular JIA, even when their disease was inactive [48]. Indeed, hierachical clustering analysis, a method for asking, "Which group is most like the other group?"scattered patients with active and inactive disease indistinguishably, as shown in Figure 2. Even more interesting were *in silico *models of the differentially-expressed genes (Figure 3), which showed clusters of IFNγ and IL-8-related genes, the significance of which will be explained shortly. Subsequent work from our laboratory has shown that these expression abnormalities persist even when children fit consensus criteria for remission (unpublished data). This contrasts with what we have reported in peripheral blood mononuclear cells of children with JIA, where gene expression profiles normalize after therapy [49]. The obvious question is, "What is this defect, and what can we do about it?"

### Neutrophils as oscillators

The key as to where the defect in JIA/JRA neutrophils might lie emerged from the *in silico *modeling of the gene expression data. As noted above, computer modeling of the functional relationships between the different genes suggested linked networks of IL-8 and IFNγ-regulated genes. The significance of this finding may lie in the underlying dynamic properties of cell chemistry.

Oscillations are found throughout nature, from bacteria to man [50]. Oscillations show three characteristics: frequency, amplitude, and phase; examples of these are the heart rate, stroke volume, and relative timing of the opening of the heart valves. In addition to this macroscopic example, oscillations in calcium signaling, mitosis, and hormone action have been studied.

Neutrophil oscillations were first reported in the late 1980s (e.g., Jager et al [51]). Since that time, a broad variety of neutrophil oscillations has been reported and their relationship to human disease documented [52]. These oscillations are not peripheral events in cell behavior, but, rather, are a central element of neutrophil activation, and therefore potentially critical to our understanding human inflammatory diseases.

Renewed interest in the phenomenon of neutrophil oscillations began with observations demonstrating physical proximity between β_2 _integrins and other cell-surface receptors, which allowed the integrins to serve as signal transducers for glycophosphatidylinositol (GPI)-linked proteins. Furthermore, when receptor proximity on living cells (e.g., between the complement receptor CR4 and urokinase plasminogen activator receptor [uPAR]) was measured with resonance energy transfer microscopy, it was found to oscillate in time[53]. The fact that all of the CR4 and uPAR molecules either come into proximity or move away from each other simultaneously implies biological coherence in receptor action; the receptors are not behaving as individual signaling units but as a collective. Moreover, the period of CR4-uPAR oscillation was similar to that reported previously by other research groups for other neutrophil oscillators. Furthermore, the shape of the oscillatory waves could be modulated by suboptimal doses of kinase and phosphatase inhibitors, indicating that the dynamic uPAR-CR4 interactions are coupled with oscillatory signal transduction machinery. This suggests that the structural and functional oscillations of neutrophils are tied to the same fundamental chemical oscillator. The heterogeneity of the oscillators, such as receptor properties, membrane potential, shape, actin, etc would simply reflect the experimental readouts employed.

Subsequent work in the Petty laboratory has directly linked cell metabolism, oscillatory phenomena, and cell signaling [54]. This has been achieved by measuring fluctuations in NAD(P)H levels, which respond parallel to, but 180° out of phase, with ATP. Furthermore, because NAD(P)H is fluorescent, fluctuations can be measured without perturbing the cells (although NADH and NADPH cannot be distinguished spectroscopically). Subsequent work in the Petty laboratory using NAD(P)H autofluorescence has shown that (1) metabolic oscillations drive receptor signaling and receptor oscillations; (2) variations in NAD(P)H amplitude and frequency are carried "downstream" to other functional, oscillatory phenomena such as superoxide and nitric oxide production; (3) the period of NAD(P)H oscillations is tightly linked to the key event in neutrophil activation, glucose influx.

A wide variety of biological materials are known to influence intracellular calcium signaling. Similarly, this group has discovered numerous materials that influence the amplitude or frequency of calcium and/or metabolic oscillations. These include fMLP, immune complexes, IFNγ, TNFα, and others [55,56]. It is important to note that specific agents regulate specific aspects of the oscillatory phenomena. Specifically, wave amplitude and wave frequency are regulated independently, and the mechanisms that regulate each are stringently insulated from one-another. Some of the relevant mediators can be summarized as follows:

1. IFNγ and PMA trigger amplitude modulation of the oscillations.

2. TNFα, IL-8, fMLP, and immune complexes cause calcium and metabolic oscillations to double in frequency.

3. Other biological response modifiers, including IL-2 and IL-6, double oscillation frequency, but only during concurrent amplitude modulation, e.g., with IFNγ.

**Figure 4:**
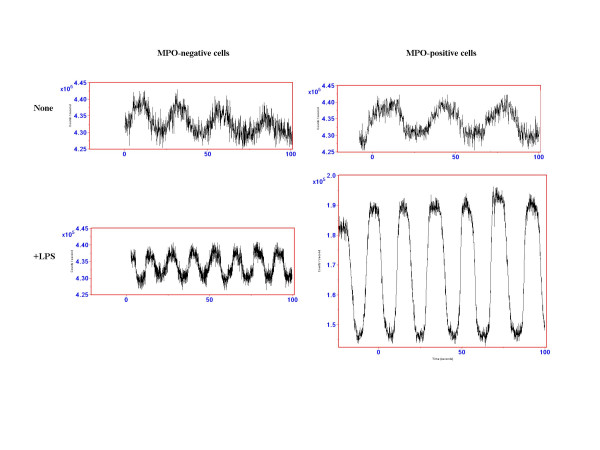
Aberrant patterns of neutrophil NAD(P)H oscillation in JRA neutrophils. JRA patients express a small population (10–30% of the cells) that are MPO-positive and show both frequency and amplitude enhancement of NAD(P)H oscillations triggered by frequency-modulating LPS. These findings demonstrate a breakdown in the fundamental metabolic pathways regulating neutrophil oscillatory activities in JRA patients. From Jarvis et al, *Arthritis Res Therapy *2006. The authors retain the copyright for this material.

With this as a background, we get back to interpreting the gene expression profile in JRA neutrophils. The patterns of IL-8 and IFNγ-regulated gene expression suggested that it would be fruitful to examine other fundamental cellular processes regulated by those mediators, specifically NAD(P)H oscillations. The findings suggested a fundamental breakdown of the processes that insulate frequency and amplitude regulation in JRA neutrophils, as shown in Figure 4. That is, agents that normally evoke only increases in NAD(P)H frequency (LPS, immune complexes), induced both frequency *and *amplitude enhancement in a subset of JRA neutrophils. This defect was observed in patients with both active and inactive disease. Thus, we assume that this defect may be primary, although the possibility that it is caused or driven by more fundamental aberrations in adaptive immunity cannot be excluded.

### Redefining the pathogenic paradigm and exploring the future

It is time to ask whether the T cell deserves the place it has held for so long in the center of JIA pathogenic paradigms. While there is insufficient data to replace the central place of the T cell with the neutrophil (although Figure 1 accounts for the known data as well to us than the T cell centered paradigm), it is time to give neutrophils their due place. New data from multiple laboratories, using techniques as diverse as genomics, *in silico *modeling, and single-cell microscopy, all suggest that we have overlooked the critical role neutrophils may play in JIA pathogenesis. Furthermore, these data account for observed phenomena not explainable by T cell-centered hypotheses (e.g., the applicability of S100 proteins as biomarkers of response). Furthermore, they account for the known involvement of adaptive immunity far better than T cell-centered hypotheses account for the known involvement of the innate immune system, although there is reason to believe the interaction is bi-directional [57]. Indeed, the efficacy of anti-inflammatory therapies, such as anakinra [58], MRA [59], thalidomide[60] (a powerful suppressor of neutrophil function), as well as T cell inhibitory therapy (abatacept, cyclosporine) suggests that distorted cross-talk innate between adaptive immunity is a critical element in JIA pathogenesis. We would simply argue that this does not necessarily imply that the dysfunctional relationship *starts *with T cells.

There is still much to do. It is very likely that we will need rigorous applications of systems biology approaches and ongoing cooperation between laboratories invested in both innate and adaptive immunity to unravel the complexities that are only now coming into focus. It is clear that we know little of the molecular basis of disease pathology and nothing of the etiology of any of the forms of JIA. Maybe a short moratorium on the word, "autoimmune," as applied to this family of diseases, is in order, since use of the word suggests that we already know *where *the relevant immunopathogenic events may reside. Above all, we need to be bold and creative in our thinking, ready to cut loose from comfortable paradigms, humble in admission of what we still don't know, and persistent in our determination to get better answers for hundreds of thousands of children and young adults with arthritis.
